# Linear Registration of Brain MRI Using Knowledge-Based Multiple Intermediator Libraries

**DOI:** 10.3389/fnins.2019.00909

**Published:** 2019-09-11

**Authors:** Xinyuan Zhang, Yanqiu Feng, Wufan Chen, Xin Li, Andreia V. Faria, Qianjin Feng, Susumu Mori

**Affiliations:** ^1^School of Biomedical Engineering, Southern Medical University, Guangzhou, China; ^2^Guangdong Provincial Key Laboratory of Medical Image Processing, Southern Medical University, Guangzhou, China; ^3^Department of Radiology, School of Medicine, Johns Hopkins University, Washington, ME, United States

**Keywords:** linear registration, mediator selection, T1-weighted brain image, MNI space, dice value

## Abstract

Linear registration is often the crucial first step for various types of image analysis. Although this is mathematically simple, failure is not uncommon. When investigating the brain by magnetic resonance imaging (MRI), the brain is the target organ for registration but the existence of other tissues, in addition to a variety of fields of view, different brain locations, orientations and anatomical features, poses some serious fundamental challenges. Consequently, a number of different algorithms have been put forward to minimize potential errors. In the present study, we tested a knowledge-based approach that can be combined with any form of registration algorithm. This approach consisted of a library of intermediate images (mediators) with known transformation to the target image. Test images were first registered to all mediators and the best mediator was selected to ensure optimum registration to the target. In order to select the best mediator, we evaluated two similarity criteria: the sum of squared differences and mutual information. This approach was applied to 48 mediators and 96 test images. In order to reduce one of the main drawbacks of the approach, increased computation time, we reduced the size of the library by clustering. Our results indicated clear improvement in registration accuracy.

## Introduction

Image registration is a method used to align multiple images to ensure the spatial correspondence of anatomy across different images. There are two types of registration algorithm, which are based on transformation models: linear and non-linear registration. Linear registration is used widely and predominantly involves six-parametric rigid transformation (rotation and translation on x, y, and z coordinate axes) or 12-parametric affine transformation (rotation, translation, scaling, and shearing on x, y, and z coordinate axes). The linear registration is global in nature while non-linear registration has a higher degree of elasticity which can model local deformation.

Registration is an essential step for many types of medical image analysis including voxel-based analysis, change detection, cross-modality image fusion and image segmentation [for reviews see Gholipour et al. (2007), Despotovic et al. (2015)]. The success of studies involving image analysis depends heavily upon registration accuracy. In general, linear registration is an essential first step for registration-based analysis, followed by local non-linear registration. Therefore, the quality of the initial linear registration is often crucial for subsequent steps.

Although it is theoretically simple, in reality, initial linear registration can be one of the most difficult steps to perform. This is because a number of factors exert influence and could lead to the gross failure to align two images; such factors include different tissue locations and orientations, and the area of tissue covered by the images. The initial registration is, to some degree, ill-posed because the computer algorithms used do not have *a priori* information relating to the locations of the target organs. However, these algorithms are asked to align only the target organs, regardless of whether there are any additional or missing structures in one of the images. For example, if one of the images has a larger field of view and contains additional tissues (for example, data relating to the neck when carrying out brain MRI), then complications may arise when aligning the target organ, particularly when their initial locations and rotations are significantly different when compared between the two images.

To minimize this problem, various types of methods have been proposed for the cost functions and optimizers. For example, many intensity-based similarity metrics have been proposed in order to increase the accuracy of alignment, such as mutual information (MI) (Maes et al., 1997), correlation coefficient (CC) (Kim and Fessler, 2004), ratio image uniformity (RIU) (Woods et al., 1998a, b), Kullback-Leibler divergence (Konukoglu et al., 2011), and residual complexity (RC) (Myronenko and Song, 2010; Zhang et al., 2013). Different optimization methods, including the gradient descent optimization method (Rueckert et al., 1999; Myronenko and Song, 2010; Qiao et al., 2016) and the Levenberg–Marquardt optimization method (Fitzgibbon, 2003; Gaidhane et al., 2012) have become widely used. Iterative methods are also commonly used, in which the degree of freedom for image transformation, smoothing, and down-sampling factors are systematically changed (Nestares and Heeger, 2000). Nonetheless, it is common that alignment failures still occur occasionally. To reduce the chance of failure, certain recommendations have been published for widely used tools. For example, Automated Image Registration (AIR) recommends the manual removal of non-brain tissues (Woods et al., 1998a, b).

In this paper, we propose a knowledge-based approach to improve registration accuracy, which can be combined with any form of registration algorithms. A key component of this approach is a collection of MR images, referred to as “mediator images,” which serve as intermediates between a template and subject images. These mediator images were pre-processed to identify their transformations into template images (e.g., MNI space) using linear registration facilitated by manually placed landmarks. If the subject image could be accurately registered to one of these mediator images, then a combination of linear transformation from subject image to the mediator image, and pre-determined transformation from the mediator to the template in the MNI space, can be applied for the subject image. In this study, we used affine transformation based on the commonly used AIR package. We evaluated our proposed method against conventional direct registration between the subjects and the template. In addition, we tested a strategy to construct an effective collection of intermediate images.

## Materials and Methods

### Data Sources

#### Template Atlas in the MNI Space

A T1-weighted (MPRAGE) image from the JHU-MNI-SS atlas was used as the template image, and was based on data from a single-subject, as described previously (Oishi et al., 2009). The atlas matrix was 181 × 217 × 181 with a resolution of 1 mm × 1 mm × 1 mm.

#### Test Data and Mediators

Data were acquired from multiple scanner vendors (GE, Phillips, and Siemens) and field strengths (1.5 T and 3 T), using an MPRAGE sequence with site-dependent scan protocols. A portion of the data were obtained from the ADNI database (adni.loni.usc.edu). The ADNI was launched in 2003 as a public–private partnership, led by Principal Investigator, Michael W. Weiner, MD. The primary goal of the ADNI database is to assess whether serial MRI, positron emission tomography, other biological markers, or clinical and neuropsychological assessments can be combined to measure the progression of mild cognitive impairment and early Alzheimer’s disease. For the data acquired in JHU, the Johns Hopkins University IRB committee reviewed and approved the study protocol.

The image resolutions were 1 × 1 × 1 or 1 × 1 × 1.2 with varying matrix sizes across different subjects. All scans were acquired in the sagittal orientation, which tend to have much larger fields of view than that of the MNI template. The brain locations and orientations are also highly variable in this dataset. Thus, linear registration of these data to MNI templates are challenging. In this study, test images were registered to the template using the single-mediator method described in this paper. All results were then visually inspected and failed cases were re-registered using manually placed landmarks and DiffeoMap software^1^ (see section “Results,” for the failure rate). These images were further processed through MRICloud^2^ to automatically segment the brain. The segmentation quality was visually inspected and cases with segmentation errors were removed from the results. In the final dataset, 144 T1-weighted images were used. These images were randomly divided into two groups: 96 subjects were used as test data and 48 were used as mediators.

### Study Methods

#### Registration Algorithm

Image registration was performed using the 12-parameter affine transformation, the AIR package and trilinear interpolation (Woods et al., 1998a). Other parameters were set to default values, including cost function (the ratio image uniformity).

In this study, AIR was chosen because it was one of the first and most widely used software packages for linear registration of MR images. While we currently have a wide variety of choices for linear registration tools with advanced features such as combinations of different levels of downsampling, smoothing, and modes, the simplicity of AIR algorithms makes it suitable as a benchmark for this study.

#### Measurement of Registration Quality

Registration quality was measured by calculating the degree of overlap between two binary brain masks. The 96 test images were previously registered with the MNI template and then segmented. Therefore, their brain mask (gray matter + white matter + ventricles) in the MNI space could be used to measure registration accuracy. To quantitatively evaluate the registration, we used the Dice coefficient to measure the spatial overlap between brain masks created from the template and automatically registered test data. The Dice coefficient was defined as follows (Dice, 1945):

$$\mathrm{Dice}=\frac{2\,\left|\text{X}\cap \text{Y}\right|}{\left|\text{X}\right|+\left|\text{Y}\right|}$$

X and Y represented the masked regions from the mediator and corresponding transformed subject image that is aligned to the mediator. The Dice coefficient ranges from 0 to 1. The higher the Dice coefficient, the better the registration. In extreme cases, the Dice coefficient is 0 (when there is no overlap between two compared regions) or 1 (for complete overlap). Dice coefficients are most widely used to measure the degree of overlaps in the field of neuroanatomy and registration is commonly considered to be satisfactory when the Dice coefficient is larger than 0.85. Thus, in this study, a Dice coefficient > 0.85 was considered as good alignment while a Dice coefficient < 0.85 was considered to be poor alignment.

#### Pairwise Direct Linear Registration Between Subject and Template

**FIGURE 1:**
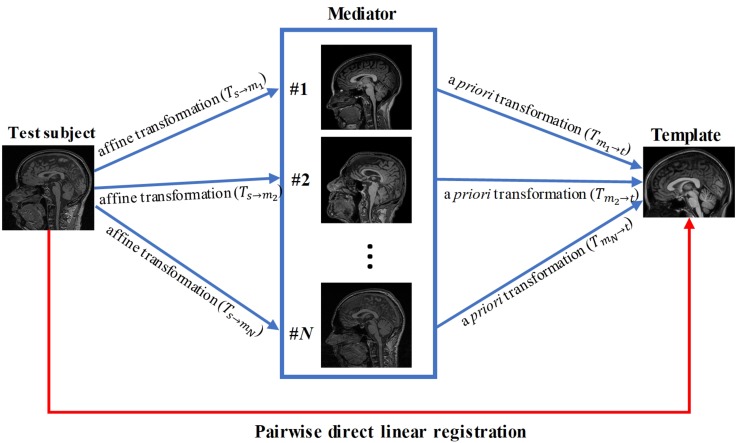
Schematic diagram of our knowledge-based registration method.

Pairwise direct linear registration represents the performance of the conventional approach. It was performed by directly aligning data from each subject (96 test data) to the MNI template using the affine transformation, as illustrated by the red arrowed line in Figure 1. Transformations (*T*_s←t_) were obtained using AIR, which was applied to the brain mask of each subject for Dice coefficient measurement.

#### Linear Registration With a Single Mediator

We tested if a mediator with similar imaging parameter could improve the registration accuracy compared to the pairwise direct linear registration. A mediator was arbitrary selected from the 48 mediators and performance of the single-mediator approach was evaluated. The affine transformation was performed to register each test subject to the single mediator and the transformation *T*_s←m_) was then obtained. The composition *T*_s←m←t_) of the transformations between the *T*_s←m_ and *T*_m←t_ was performed to transform the test image and the associated brain mask into the template space, as follows:

$$T_{s\to m\to t}=T_{s\to m}\circ T_{m\to t}$$

where the symbol ° denotes the composition of the two transformation fields. The brain mask, transformed to the MNI space, was then used to calculate the Dice coefficient.

#### Linear Registration With Multiple Mediators

Forty-eight brain images were used as mediators. Affine transformation was first performed to register the test images to each mediator and the transformations (*T*_s→m_i__,*i* = 1,2,⋯,48) were then obtained. The composition *T*_s←m^* ←t_ of the transformation *T*_s←m^*_ and *T*_m^* ←t_ was performed to transform the test image and the associated labels into template space, similar to the single mediator case:

$$T_{s\to m^{*}\to t}=T_{s\to m^{*}}\circ T_{m^{*}\to t}$$

where *m*^∗^ represents the best mediator with highest Dice coefficient. In the ideal situation, we have the knowledge about the best mediator for a given test image. In this study, the availability of the brain mask in the MNI space for the test data allowed us to identify the best mediator; namely, the mediator with the highest Dice coefficient was considered as the “true” best mediator. However, it is important to consider that the best mediator will be unknown in practical situations where brain masks are not available and the Dice coefficient cannot be calculated.

The 144 subject images (96 test images and 48 mediators), the affine transformation matrices from each test image to mediators *T*_s→m_i__,*i* = 1,2,⋯,48), the pre-determined linear transformation matrices from mediators to MNI space (*T*_m_i_→t_,*i* = 1,2,⋯,48) and the experimental results can be found in https://doi.org/10.5281/zenodo.3360488.

#### Strategy for Selecting the Best Mediator

To estimate the best mediator, we tested the sum of squared difference (SSD) and mutual information (MI). Both SSD and MI criteria were calculated using voxel intensity information from the whole image following registration between the test and mediator images.

Sum of squared difference is based on the difference in intensity when compared between each mediator and the registered subject image, and is defined as follows:

$$\text{SSD}\,\left(X,Y\right)=\sum_{\text{i}}{\left(X_{\text{i}}-Y_{\text{i}}\right)}^{2}$$

where *X*_i_ and *Y* represent the intensity value of the *ith* voxel of mediator *X* and the registered data *Y*. The smaller the SSD value, the better the registration. The mediator with the smallest SSD was selected as the best mediator. Each mediator was normalized for intensity to the target image using histogram matching prior to the calculation of SSD.

Unlike SSD, MI does not require image intensity matching between the two images. Rather, MI is a measure of the informational correlation between images *X* and *Y*, and is calculated as follows:

$$\text{MI}\,\left(X,Y\right)=\sum_{x,y}p_{X,Y}\,\left(x,y\right)\,\log\frac{p_{X,Y}\,\left(x,y\right)}{p_{\text{X}}\,\left(x\right)\,p_{\text{Y}}\,\left(y\right)}$$

where *p*_X,Y_(*x*,*y*) is the joint distribution of the voxel intensities of images *X* and *Y*. *p*_x_(*x*) and *p*_Y_(*y*) are the marginal distributions of images *X* and *Y*. MI is based on the assumption that there is a maximum correlation between the voxel intensities of the images when they are correctly aligned. MI value range from 0 to 1. The mediator with the highest MI value was selected as the best mediator. An important note to consider is that the best mediator depends on the subject and may differ between different subjects.

#### Mediator Shrinkage by Clustering

In order to use the multi-mediator approach with a certain number (*N*) of mediators, we need to perform registration *N* times; the computing time increases proportionally as the number of mediators increases. Among the 48 mediators, some mediators may carry redundant information. To reduce the computation time, with minimum loss of the accuracy, we tested mediator shrinkage by clustering based on similarity matrices. The similarity criterion was based on inter-registration among the 48 mediators, from which SSD values were calculated as the criteria. The clustering analysis was conducted by the pheatmap package in R.

## Results

### The Performance of Pairwise Direct Linear Registration

**FIGURE 2:**
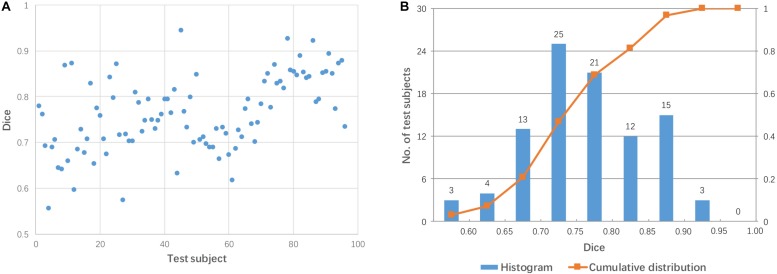
Dice coefficients obtained by pairwise direct linear registration between test and template images. **(A)** Dot plot of the Dice coefficients against 96 test images; **(B)** Histogram of the Dice coefficients with cumulative percentage.

Each test image (from 96 subjects) was aligned to the template directly using affine transformation. The accuracy of the pairwise direct registration was measured by Dice overlap of the brain masks. Figure 2 shows the Dice coefficient for the pairwise linear registration across 96 test images. The Dice coefficients ranged from 0.556 to 0.945 with a mean of 0.764 ± 0.08. As seen from Figure 2B, the Dice coefficients of most test images are located between 0.65 and 0.9, and only 18 test images achieved a good alignment with a success rate of 19% based on the specific criterion used (Dice coefficient > 0.85).

**FIGURE 3:**
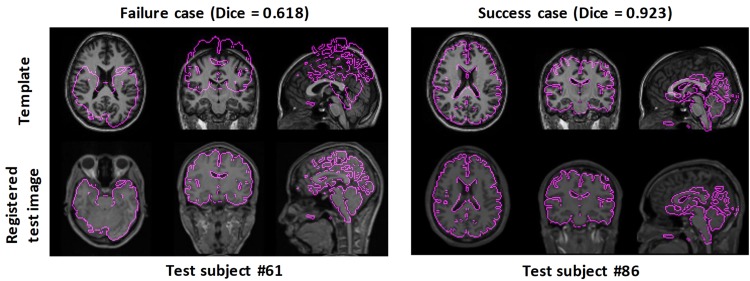
Examples of failed and successful pairwise linear registration between test and template images. Top row: template image. Bottom row: registered test images. For visual clues, the brain masks of the registered test images were overlaid onto the template image.

Figure 3 shows two examples: a failed case and a successful case. To further facilitate visual comparison, brain masks were superimposed on the images. We found that test #61 was misaligned to the template while test #86 was aligned successfully.

### The Performance of Linear Registration With a Single Mediator

**FIGURE 4:**
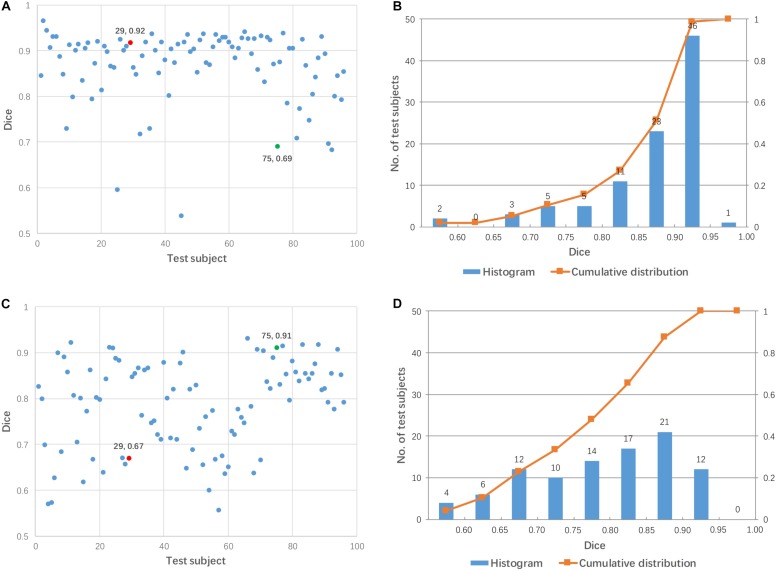
Dice coefficients obtained by linear registration using two arbitrary mediators for 96 test subjects. **(A,B)** Mediator #1; **(C,D)** mediator #17. Cases #29 and #75 are shown by red and green colors, respectively.

Linear registration was used to align 96 test images to each mediator (48 mediators in total). Figure 4 shows the Dice coefficients from 96 test images for two arbitrary-selected mediators. As shown in Figure 4, the same mediator resulted in different registration performance across different subjects, while different mediators produced different registration performance for the 96 test subjects. For example, mediator #1 was a good intermediate image for subject #29 (red dot in Figure 4A) but was poor for subject #75 (green dot). On the other hand, mediator #17 was excellent for subject #75 but poor for subject #29. Overall, mediator #1 produced a better performance of linear registration, compared to mediator #17 which is clearly illustrated in Figure 4B. Across the 48 mediators, the average Dice coefficient was 0.840 with a 55% success rate (Dice > 0.85). Compared to pairwise direct linear registration, the single mediator approach improved the mean Dice coefficient and success rate.

### The Performance of Linear Registration With Multiple Mediators

**FIGURE 5:**
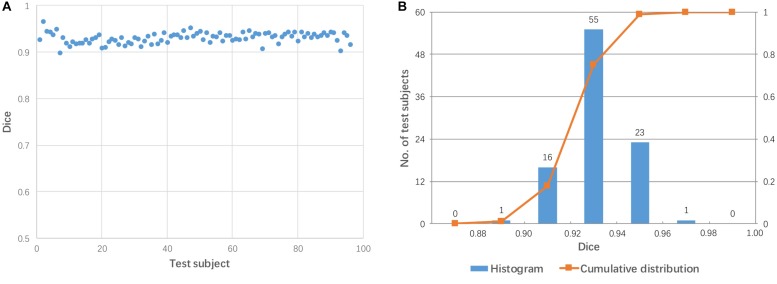
The highest Dice coefficient among the 48 mediators used for each subject. **(A)** Dot plot of the Dice coefficients against 96 test images; **(B)** histogram of the Dice coefficients with cumulative percentage.

For each subject, the mediator with the highest Dice coefficient after linear transformation was considered to be the best mediator. Figure 5 shows the performance of linear registration using the best mediator for each test subject. The Dice coefficient ranged from 0.900 to 0.966 with a mean of 0.931. The Dice coefficients are centrally located between 0.90 and 0.96 as shown in Figure 5B. Thus, it was possible to achieve successful registration for all subjects if the best mediator for each subject was selected. Note that none of the Dice coefficients among 96 subjects is larger than 0.98 which can be well explained by that linear registration can not change the brain shape and perform a perfect alignment between mediator and subject.

**FIGURE 6:**
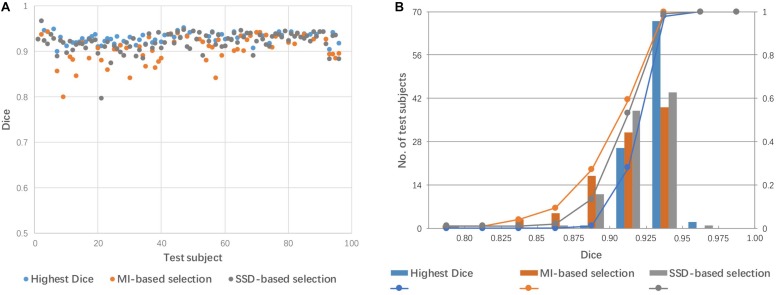
Dice coefficients obtained by linear registration with the best mediator, as selected by SSD and MI criteria. **(A)** Dot plot of the Dice coefficients against 96 test images; **(B)** histogram of the Dice coefficients with cumulative percentage.

Figure 6 shows a comparison of the performance of linear registration with the best selected mediator, based on SSD and MI. The rate of success alignment (Dice coefficient > 0.85) is 99% and 96% for SSD and MI-based selection criteria. The mean Dice coefficients were 0.921 and 0.912 for SSD and MI, respectively.

### The Performance of Mediator Reduction by Clustering

**FIGURE 7:**
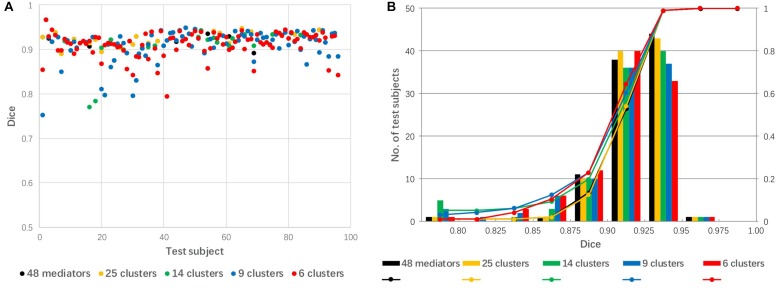
The effect of mediator reduction on Dice coefficients. **(A)** Dot plot of the Dice coefficients against 96 test images; **(B)** histogram of the Dice coefficients with cumulative percentage.

The 48 mediators were clustered into 25, 14, 9, and 6 groups based on the similarity among these groups. Figure 7 shows a comparison of linear registration performance using the 48 mediators along with number-reduced mediators. The mean Dice coefficients were 0.921, 0.920, 0.911, 0.910, and 0.911, while the success rates were 99, 99, 94, 94, and 96% for the 48 mediators and the reduced mediators (25, 16, 9, and 6), respectively. The registration accuracy was slightly reduced with the 25 mediators (approximately reduced by 2) and there was a further reduction to a 4–5% failure rate for 14 mediators and less.

## Discussion

### The Role of Linear Registration and Potential Problems

Linear registration is often the first step for various forms of image analysis and, thus, the quality of subsequent processing steps are heavily influenced by the accuracy of the linear registration step. Although linear registration is a theoretically simple step, it can be associated with a range of practical challenges due to unpredictability in the morphological and spatial variability of test data relative to the template to which the data are registered.

There have been numerous algorithms proposed to minimize the risk of failure in this initial alignment process. Probably one of the most widely used approaches is to perform iterations with varying degrees of down-sampling, smoothing factors, and the degree of freedom for the transformation (3–12 modes). As a publicly available resource, the Insight Tool Kit (ITK) has been extensively used in many image analysis tools (Johnson et al., 2018). Currently, example codes from ITK libraries provide 12 different choices for linear registrations; as the algorithms become more advanced, the number of parameters to optimize also increases. The ITK guidebook reports that there are “numerous parameters involved in tuning a registration method for a particular application” and “it is not uncommon for the registration process to run for several minutes and still produce a useless result” (Johnson et al., 2018).

Figure 2 shows 96 test images; of these, only 18 test images achieved good alignment (Dice coefficient > 0.85) to the template. This high failure rate (81%) was somewhat exaggerated because of two factors. First, we employed a simple registration algorithm without iterative approaches. Second, there were large geometrical differences between the test data and the template (the MNI coordinate space); the test data were obtained from widely used sagittal protocols, which tend to have much larger fields-of-view than that of the MNI space template. Furthermore, brain positions and orientations were not acquired using consistent criteria in this heterogeneous dataset. Finding an appropriate alignment to the MNI space is a challenging task for automated algorithms. This baseline data is, however, suitable to examine the improvement of registration accuracy by employing multiple mediators.

### Knowledge-Based Approach

In the approach described herein, improvement of the registration algorithm was not the prime target of the research. Rather, our goal was to use multiple mediators, which can be considered as a knowledge-based approach, because each mediator carries a pre-determined transformation matrix to register them to the MNI space. As the quality of the transformation matrices were confirmed by visual inspection, and corrected by manually placed landmarks as necessary, there was good infusion of human knowledge. One drawback of this knowledge-based approach is that it was specifically designed for sagittal scan protocols. Consequently, knowledge may need to be developed for other types of protocols that deviate significantly from typical sagittal protocols, such as coronal and axial MPRAGE scans or multi-slice spin-echo T1-weighted images. The improvements obtained by the use of mediators were obvious, even when only one mediator was utilized (Figure 4); the mean success rate using this approach improved to 55%. Using the entire mediator library (48 mediators), the success rate could potentially reach 100% (Figure 5).

### The Use of Matrices to Select Mediators

The success rate, as defined by the paper, is 100% when the best mediator was correctly chosen (Figure 5). However, in reality, there is no *a priori* knowledge relating to the best mediator. It is prudent to point out that in this study, accuracy values (Dice coefficients) could be calculated because the test images were provided from our pre-segmented atlas libraries. This enabled us to choose the best mediator using *a posteriori* DICE evaluation. Also important to note is that a high Dice coefficient cannot assure that tissues within the segmented mask are well aligned. These small misalignments between tissues can be further corrected by a following non-linear transformation. In the implementation, we selected the specified brain mask (gray matter + white matter + ventricles) to calculate the Dice coefficient. Theoretically, the segmented mask with gray matter, white matter and ventricles has large segment size which is more suitable for the alignment evaluation (Taha and Hanbury, 2015). By visual inspection, we found that the registered subject image with high Dice coefficient has a good overall alignment to the mediator and vice versa. Besides the Dice coefficient, the distance-based metrics such as mahalanobis distance and probabilistic distance can be considered as an alternative measurement to evaluate the registration (Taha and Hanbury, 2015) which is beyond the scope of the current study. In reality, the performance of the multi-mediator approach hinges upon a similarity metric in order to select the best mediator. In the present study, we tested SSD and MI, which are widely used as cost functions to estimate the goodness of the alignments. We found that SSD showed a higher performance (99 vs. 96% of success rate), although, neither SSD nor MI could achieve the best performance based on *a posteriori* mediator selection. The performance of intensity-based metrics could be influenced by the spatially varying intensity heterogeneity across varying mediators. Consequently, the methodology used to select the best similarity measure, still needs further improvement. The mediator selection can also considered as finding the nearest neighbor of the subject image with a defined distance function which is basically “1-nearest-neighbor classification” (Kumar et al., 2008). Along the line of this concept, more elaborated approach could be tested to improve the final registration performance. For example, we could borrow the idea of *k*-nearest neighbors classification algorithm (Belongie et al., 2002; Weinberger and Saul, 2009) in which *k* similar mediators with nearest neighbors to the subject image are selected and the corresponding *k* combined transformations with weighted averaging are used as the final transformation from the subject image to MNI space. In addition, the deep learning-based classification, such as convolutional neural network (Krizhevsky et al., 2017; Sultana et al., 2018), could be adapted to predict the best mediator more accurately which generally needs vast amounts of data with classification labels.

The idea of selecting appropriate atlases from an atlas library based on similarity measures is widely used for multi-atlas segmentation techniques (Wang et al., 2013a, b). Our approach should be considered as an extension of previous work within the context of initial linear alignment (i.e., mediator selection).

Recently, similar strategies have been reported, which also utilize a mediator to improve the accuracy of image registration. For example, several learning-based registration methods have been proposed to predict the initial deformation field (Tang et al., 2009; Kim et al., 2012, 2015); in such methods, the estimated initial deformation is applied onto the template image to create a deformed template as an intermediate image which is close to the subject. Thus, the registration between the subject and template is reduced to the simple problem of registering the subject to the estimated intermediate template. The creation of a synthesized mediator such as this could represent an alternative way to our simpler mediator library approach.

### Computation Time and the Number of Mediators

The proposed knowledge-based method requires the user to repeat linear registration for each mediator. Therefore, computation cost could represent one of the drawbacks of this approach. For example, we collected 48 subject images as mediators in which linear registration needed to be repeated 48 times for each test data. In our research, affine linear registration by the AIR package took 6 min for the 48 mediators using a Windows 10 computer equipped with an Intel(R) Xeon(R) CPU 2.00 GHz (2 processors) and 16 GB RAM. Because the linear registration of each mediator to the test image is independent, parallel computing could significantly accelerate the linear registration process.

In general, increasing the number of mediators is expected to lead to a lower failure rate. However, the computing time is linearly correlated with the number of mediators. In our test data, the best mediator delivered Dice coefficients in the range of 0.90–0.96 (Figure 5), which are typically close to maximum we can expect for cross-subject registration based on past publications (Aljabar et al., 2009; Chupin et al., 2009; Klein et al., 2009; Lotjonen et al., 2010; Wang and Yushkevich, 2013). Thus, the library of 48 mediators were suffice and the technical focal point was to identify the best mediator using image-based metrics such as SSD and MI values. A question that naturally follows this observation was, then, “can we achieve similar results with less mediators?” To maximize the computational efficiency and minimize the loss of accuracy, we tested mediator clustering based on cross-mediator similarity. The similarity criterion was based on the cross-mediator registration and the measurement of SSD values. In other words, if one mediator could be registered to the other with high accuracy (thus low SSD), we assumed that only one of them would be needed to represent the specific geometric features. In our research, we grouped 48 mediators into 25, 14, 9, and 6 clusters, respectively. Reduction in the number of mediators by clustering slightly reduced the registration accuracy from 48 to 25 mediators but significantly improved computing efficiency (Figure 7). Practically, the performance of the mediator library should now be evaluated using larger scale studies. Building up a robust library may also require a feedback mechanism, through which failed cases are incorporated into the library.

## Conclusion

In this study, we tested the performance of a mediator-based linear registration approach. When there was a large amount of geometrical differences between the test subject and template images, a library of mediators was deployed, which have previously defined transformation matrices to the final target space. Compared to direct registration between test and target images, the mediator-based approach substantially improved registration accuracy and reduced failure rates. The performance of this approach, however, relies on mediator-selection methods, in which a mediator was selected because it had the most similar image features to the test image. In terms of similarity measure, we tested SSD and MI. Even though both of these measures performed well, there is still room for improvement. Reducing the number of mediators by SSD-based clustering showed potential with which to maximize efficiency with minimum loss of accuracy.

## Data Availability

The datasets generated for this study are available on request to the corresponding author.

## Ethics Statement

This study was carried out in accordance with the recommendations of the Johns Hopkins University IRB with written informed consent from all subjects. All subjects gave written informed consent in accordance with the Declaration of Helsinki. The protocol was approved by the Johns Hopkins University. A portion of the data were obtained from the ADNI database (adni.loni.usc.edu). The ADNI was launched in 2003 as a public–private partnership, led by the Principal Investigator, Michael W. Weiner, MD. The primary goal of the ADNI database is to assess whether serial MRI, positron emission tomography, other biological markers, or clinical and neuropsychological assessments can be combined to measure the progression of mild cognitive impairment and early Alzheimer’s disease.

## Author Contributions

XZ wrote the manuscript, edited the code, processed the data, and plotted the figures. YF, QF, and WC revised the manuscript and analyzed the data. XL and AF edited the code and processed the data. SM supervised the study, revised the manuscript, provided the data, and interpreted the data for this work.

## Conflict of Interest Statement

SM is co-founder and CEO of “AnatomyWorks.” This arrangement is being managed by the Johns Hopkins University in accordance with its conflict-of-interest policies. The remaining authors declare that the research was conducted in the absence of any commercial or financial relationships that could be construed as a potential conflict of interest.

## Footnotes

1 www.mristudio.org

2 www.mricloud.org
